# Follicular metabolic changes and effects on oocyte quality in polycystic ovary syndrome patients

**DOI:** 10.18632/oncotarget.19058

**Published:** 2017-07-06

**Authors:** Yan Zhang, Lingyan Liu, Tai-Lang Yin, Jing Yang, Cheng-Liang Xiong

**Affiliations:** ^1^ School of Pharmaceutical Sciences, Capital Medical University, Beijing, China; ^2^ Family Planning Research Institute/Center of Reproductive Medicine, Tongji Medical College, Huazhong University of Science and Technology, Wuhan, Hubei Province, China; ^3^ Wuhan Tongji Reproductive Medicine Hospital, Wuhan, Hubei Province, China; ^4^ Reproductive Medicine Center, Renmin Hospital of Wuhan University, Wuhan, Hubei Province, China

**Keywords:** PCOS, follicular fluid, metabolomics, NMR, oocyte quality

## Abstract

Polycystic ovary syndrome (PCOS) is a common complex and heterogeneous disorder, affecting up to 10% women at reproductive age. It causes three fourth of the ovulatory infertility and PCOS patients often give poor IVF quality. Although some metabolic profiles have been investigated in PCOS patient sera and urine, the follicular fluid, providing fruitful biochemical information about oocyte environment during development has been ignored. In this work, based on NMR metabolomics approach, metabolic profile of follicular fluid of PCOS patients has been explored and compared with healthy controls. Significant increases of glycoprotein, acetate, cholesterol, significant decreases of lactic acid, glutamine, pyruvate, and alanine, have been discovered in PCOS follicular fluids. Furthermore, the Pearson correlations analysis indicated significant relationship existed between ART results and NMR detected follicular metabolites. All these results indicated that PCOS may induce dyslipidemia, low-grade inflammation, and disorder of glycolysis, pyruvate and amino acid metabolism in follicular fluids.

## INTRODUCTION

Polycystic ovary syndrome (PCOS) is a common endocrine and metabolic disorder affecting 5∼10% of women at their reproductive age [1, 2]. The clinical manifestations of this highly heterogeneous disease include amenorrhea, hirsutism, obesity, hyperinsulinemia, hyperandrogenism, polycystic ovaries via ultrasound, and it attributes three fourth of the ovulatory infertility [3]. Some researches even suggest a high risk of endometrial and ovarian cancer in PCOS [4]. In the meanwhile, oocytes collected from PCOS patients who undergo IVF are often of poor quality, leading to a high cancelation rate and low fertilization rate [5, 6]. The main underlying pathophysiological mechanism was thought to be the abnormally increased androgen and/or insulin level. The obesity, in another way, performs as a synergistic effect in PCOS patients [7]. However, the complex etiology of PCOS, as well as how PCOS affects oocytes development, relating to both environmental and genetic factors, has not been fully understood yet.

Large advances referring “omics” technologies have been explored to further understand this complex disease, which aim to set up potential molecular diagnosis means for PCOS, to discover therapy indicators for effective management of the disease, to establish some molecular “ruler” for assessing oocyte quality, etc. Amongst them, metabolomics, comprehensively investigating fingerprints of all metabolites from a specific system is considered to be promising [7, 8]. As the downstream products, metabolites summarize the final results of any biological events from both genes and proteins. Nevertheless, only a few researchers have focused on the area to discover biomarkers and explore the disease mechanism. Studies using NMR or/and GC-MS found carbohydrate, fat, and protein metabolism disturbance in PCOS plasma [9–12]. Additional abnormalities including saturated and unsaturated fatty acids, fatty acid amides, sulfated steroid, lysophosphatidylcholines, lysophosphatidyl ethanolamines, and carnitine were discovered by ultra-performance liquid chromatography coupled with mass spectrometry (UPLC-MS) [13–16]. Almost all these articles studied on human sera, except one [16] looked into urine metabolites.

Follicular fluid is a non-negligible bio-fluid that plays a crucial role in reproductive functions. The follicular fluid provides the special environment for oocyte growth. It contains metabolites that are accumulated during the process. Meanwhile, these metabolites are critical for the oocyte development. Hence the follicular fluid can provide information about biochemical status of follicle [17]. The knowledge of metabolic information from PCOS patients’ follicular fluid can provide some points of view on pathogenesis mechanisms in PCOS oocytes and how follicular metabolites affect oocytes quality in further [18]. However, follicular fluid has never been systematically studied in PCOS patients.

In this work, metabolic variance of follicular fluid from PCOS and healthy controls were discovered using ^1^H NMR metabolomics approach. The correlation between follicular metabolites and oocyte quality based on the assisted reproductive techniques (ART) results were further evaluated. In comparison with PCOS serum metabolomics studies which aimed to discover diagnostic biomarkers, our goal is to explore pathogenesis mechanisms in PCOS oocytes environment and investigate the metabolic effects on oocyte quality.

## RESULTS

### Clinical information of sample cohorts

According to the clinical diagnosis, 15 patients with PCOS and 36 healthy controls were involved in this study. The clinical information in the form of mean ± SD was shown in Supplementary Table 1. There was not any statistical difference between two sample groups in any ART results.

### Metabolic differences of follicular fluid between PCOS and controls

**Figure 1:**
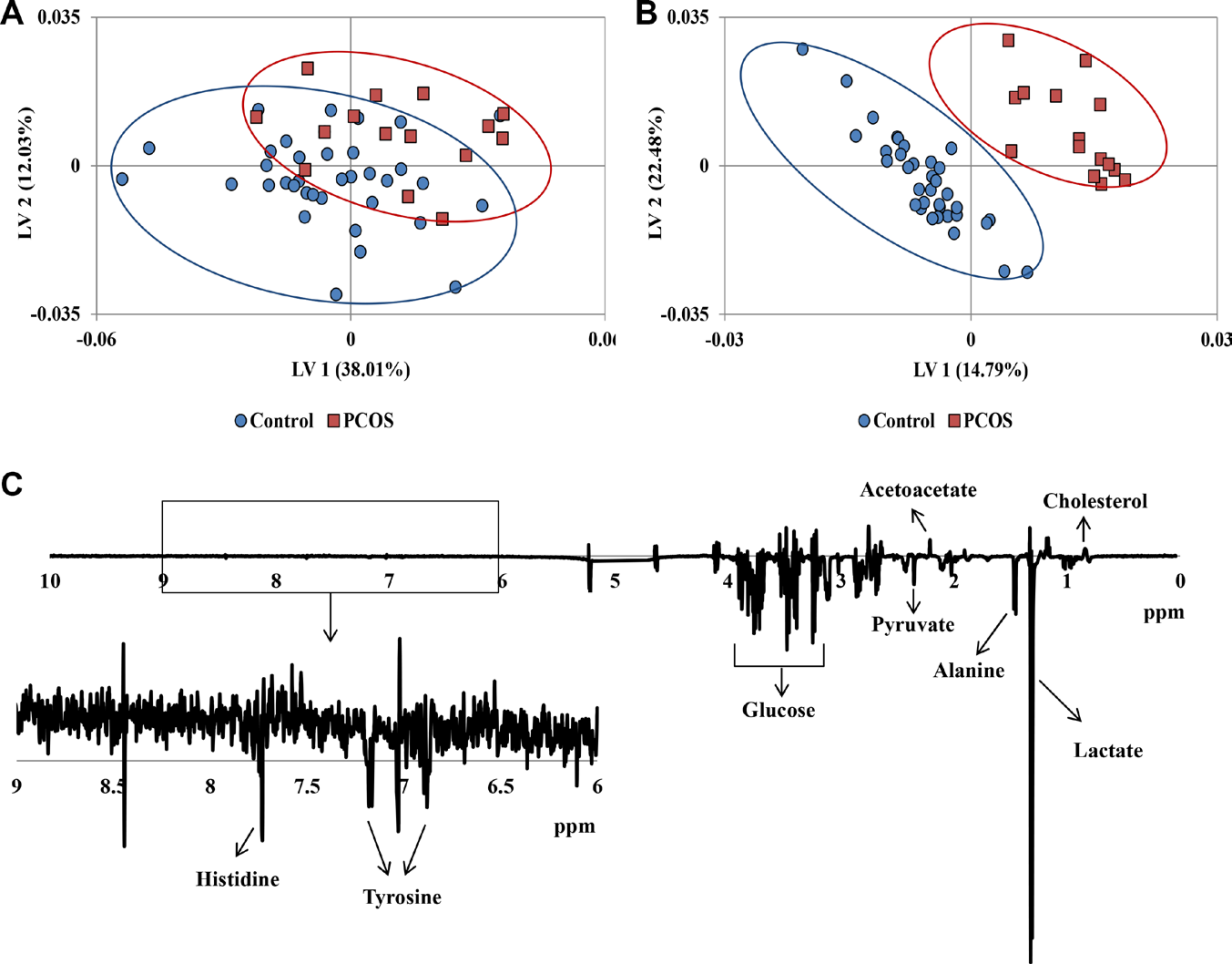
(**A**) PLS score plot of ^1^H NMR CPMG spectra of all samples. (**B**) OSC-PLS score plot of ^1^H NMR CPMG spectra of all samples. (**C**) Loadings plot on latent variable 1 (LV1) for OSC-PLS analysis of the whole CPMG spectral data.

Especially focusing on the small molecules, CPMG (Carr-Purcell-Meiboom-Gill) ^1^H NMR spectra of follicular fluid provided a large number of identifiable peaks from metabolites. The **^1^**H NMR CPMG spectra averaged over follicular fluid samples from each of the PCOS and control cohorts, together with the average difference spectrum, were shown in Supplementary Figure 1. PCOS patients and healthy controls exhibited marked differences in some metabolite signals, including alanine, lactic acid, cholesterol, and pyruvate. The dimensional reduction analysis – PLS was applied to the NMR spectra to facilitate visualization of group difference. The two population cohorts presented separated trend in PLS score plot shown in Figure 1A. The OSC-PLS further filtered out confounding physiological variations unrelated to the disease, such as diet, age, etc. Clearer separated clusters were observed in OSC-PLS score plot (Figure 1B) as it only exhibited class related biological changes. Suggested by OSC-PLS loadings that decrease of glucose, pyruvate, alanine, lactate, and increase of acetoacetate, cholesterol etc. contributed to the cluster separation along latent variable 1 (LV1, Figure 1C).

To eliminate chemical noises, a total of 59 peak regions were identified, including amino acids, organic acids, glucose, choline and lipids. The chemical shifts and multiplicities of these 59 peaks were listed in Supplementary Table 2. Integrals of respective peak regions were also obtained. Subsequent analysis was employed on identified 59 metabolite peak regions. The PLS score plot based on these 59 peak regions presented a clearer separation (Supplementary Figure 2A). As suggested by loadings, acetate (PK45), glutamine (PK37, 42), and pyruvate (PK39) contributed most to the clusters (Supplementary Figure 2B).

To focus on the actual differences between two sample groups, *P*-values were calculated by Student's *t*-test (Supplementary Table 2). A total of 9 metabolite regions with *p* < 0.05 showed the significance, including increased intensity of glycerol and lipid region, glycoprotein, acetate, cholesterol and decreased levels of lactic acid, glutamine, pyruvate, and alanine (Supplementary Figure 3). The corresponding contents of these 9 peak regions, together with another 8 regions with *p* < 0.1 were shown in the heat map (Supplementary Figure 4). The different patterns can be visually observed in PCOS from healthy controls. All of these results indicated the existence of group wise difference in follicular fluids from the two sample cohorts.

### Metabolic pathway disturbance in PCOS follicles

**Figure 2:**
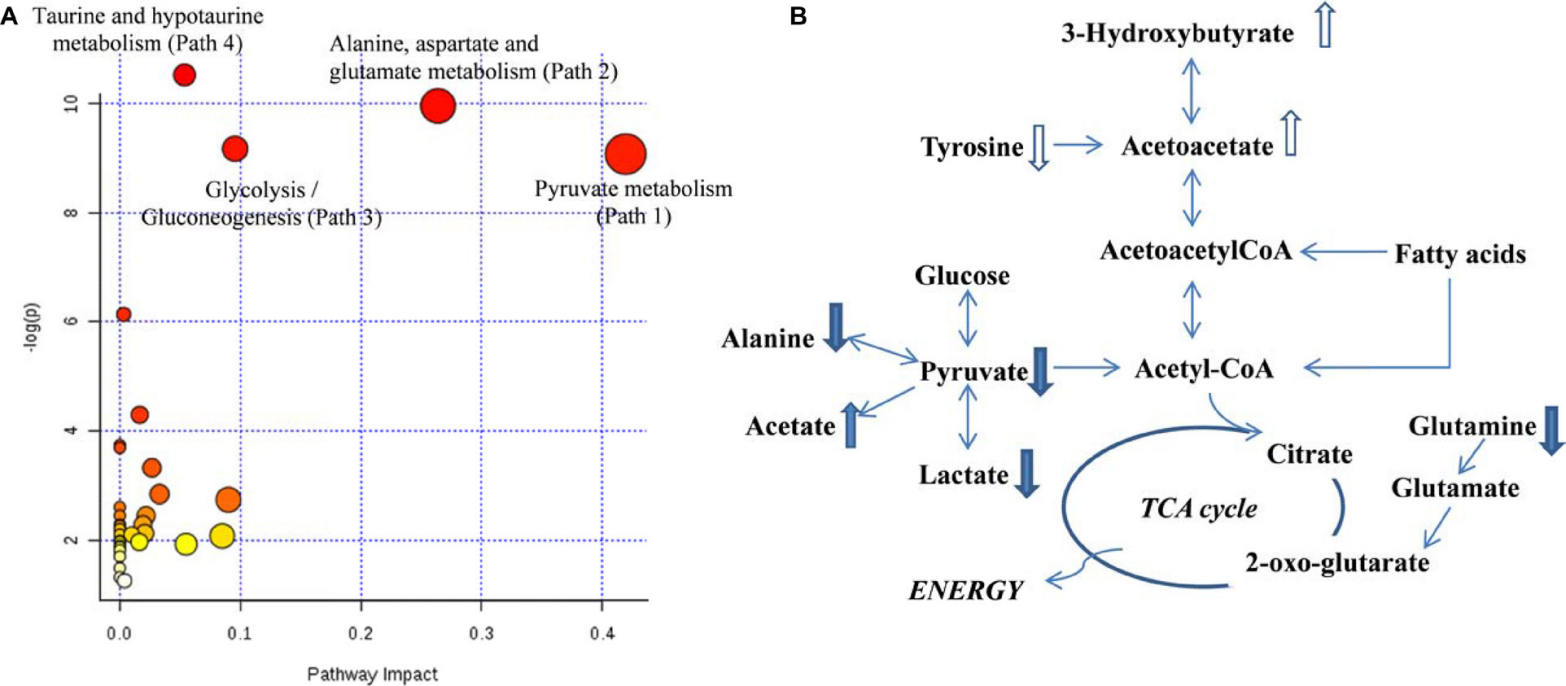
(**A**) Overview of altered metabolisms suggested by MetPA; (**B**) Pathway diagram showing altered metabolites in PCOS follicular fluids. Upward (downward) arrows indicate slightly higher (lower) levels (*p* < 0.1) in PCOS. The solid filled upward (downward) arrows indicate significantly higher (lower) levels (*p* < 0.05) in PCOS.

To explore the entire metabolic pathway map that fluctuated in PCOS follicles, all the metabolites with *p* < 0.05 between two sample cohorts were imported into MetPA. All the matching pathways were plotted in Figure 2A according to their pathway impact values of pathway topology analysis and –log (p) of pathway enrichment analysis. In addition, the metabolites with *p*-value less than 0.1 were also included here to provide more information for a more complete pathway map in Figure 2B. The affected pathways included pyruvate metabolism, glycolysis, alanine, aspartate and glutamate metabolism etc.

### Effects of metabolic perturbation on ART results

**Figure 3:**
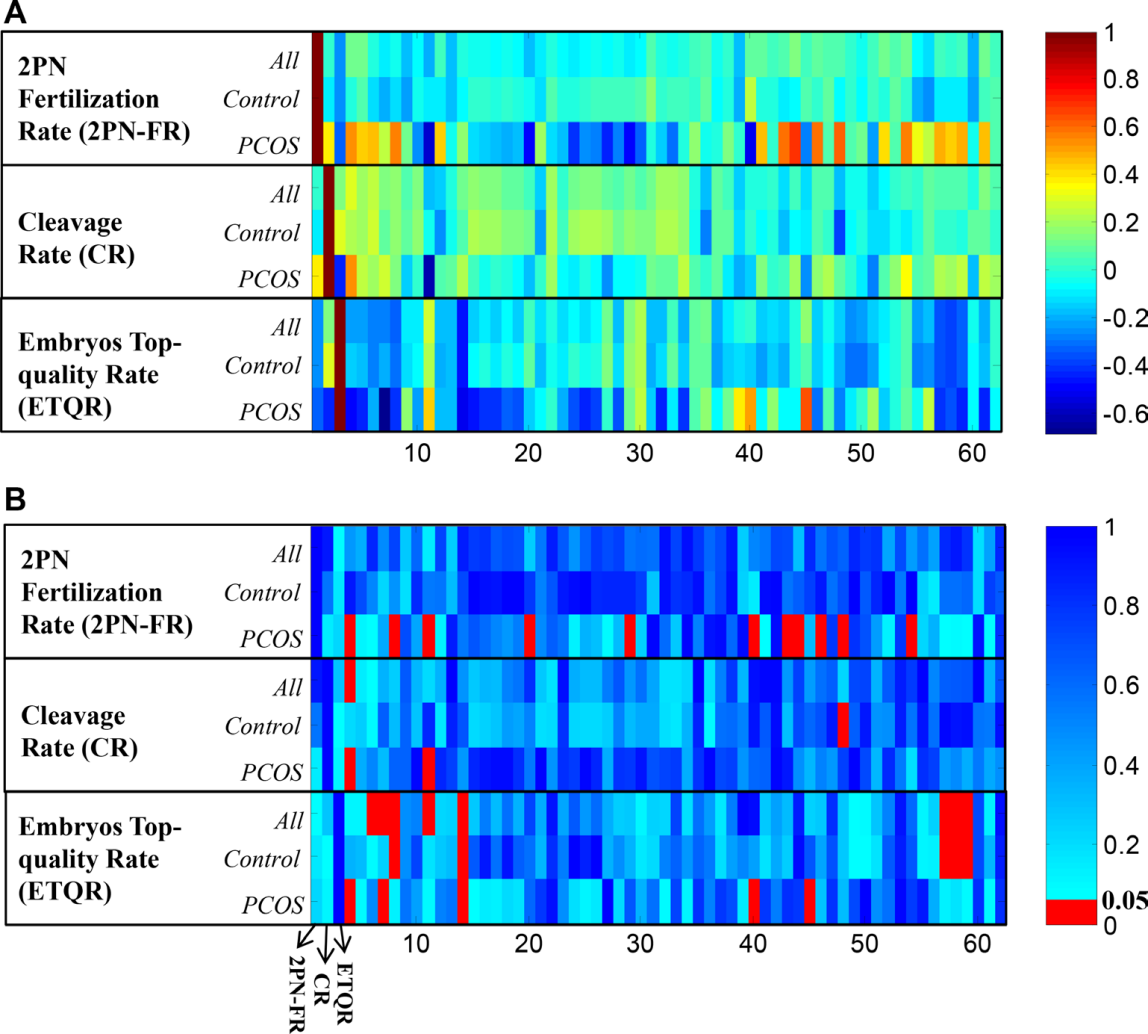
(**A**) Pearson correlation coefficients between ART clinical results (2PN fertilization rate, cleavage rate, and embryos top-quality rate) and NMR detected peak regions. The columns represent the 2PN fertilization rate, cleavage rate, top-quality embryos rate (x-axis 1∼3), and PK1 to PK59 (x-axis 4∼62) respectively; (**B**) Pearson correlation *p*-values of each paired data.

To determine whether PCOS induced metabolic disturbance has effects on ART processes, Pearson correlation was calculated between each ART variables and 59 NMR metabolite regions respectively in different sample cohorts. The correlation coefficients (Figure 3A) and their significance in *p*-value (Figure 3B) illustrated that, in PCOS patients, different metabolic patterns were correlated with ART aspects. All significant correlations in different ART aspects were summarized in Table 1.

**Table 1:** Pearson correlation coefficients between NMR detected follicular metabolites and ART results in all samples, PCOS patients, and healthy controls respectively

Peak ID	Metabolites	All	Controls	PCOS	PCOS and control *p*-value
2PN Fertilization Rate					
PK41	Acetoacetate	0.15	−0.08	0.69 (4.5E-3)	0.078
PK45	Acetate	0.07	−0.06	0.63 (0.012)	2.5E-3
PK40	3-hydroxuybutyrate	0.05	−0.10	0.60 (0.017)	0.060
PK43	Glycoprotein	0.08	0.021	0.59 (0.020)	0.020
PK8	β-glucose	−0.21	−0.10	−0.57 (0.026)	
PK1	Formate	0.11	−0.02	0.55 (0.036)	
PK5	Tyrosine	−0.05	−0.13	0.55 (0.032)	
Cleavage Rate					
PK8	β-glucose	−0.18	−0.03	–0.61 (0.016)	
PK1	Formate	0.30 (0.035)	0.21	0.55 (0.035)	
PK45	Acetate	−0.17	–0.37 (0.025)	0.09	2.5E-3
Embryos Top-quality Rate					
PK11	Creatine	–0.46 (6.7E-4)	–0.45 (5.5E-3)	–0.53 (0.044)	
PK55	Leucine	–0.38 (6.5E-3)	–0.39 (0.018)	–0.36	
PK54	Isoleucine	–0.36 (0.01)	–0.36 (0.029)	–0.40	
PK5	Tyrosine	–0.32 (0.022)	–0.31 (0.06)	–0.40	
PK4	Histidine	–0.29 (0.040)	–0.20	–0.68 (4.8E-3)	
PK42	Glutamine	0.17	0.07	0.65 (8.7E-3)	3.7E-3

In parentheses, the Pearson correlation *p*-value was illustrated if it is less than 0.05.

Significant correlation was observed in PCOS patients between their 2PN fertilization rate and several metabolites levels, including acetoacetate, acetate, 3-hydroxuybutyrate, glycoprotein, β-glucose, and formic acid. Among them, only glucose was negatively correlated with fertilization results. While, no significant correlation with 2PN fertilization rate was found in control group. Interestingly, acetoacetate, acetate, 3-hydroxuybutyrate, and glycoprotein also exhibited certain levels of significant difference between PCOS and controls.

Significantly positive and negative correlations with cleavage rate were observed in formic acid and glucose respectively in PCOS patients. Both trends were similar to those of 2PN fertilization results above. Acetic acid level was negatively correlated with the cleavage results in controls. This metabolite was also found important in fertilization step as above; however, it was positively correlated with 2PN fertilization rate in PCOS. Results showed that acetic acid plays negative role in the cleavage step in control group, while it plays positive role in the fertilization step in PCOS group.

**Figure 4:**
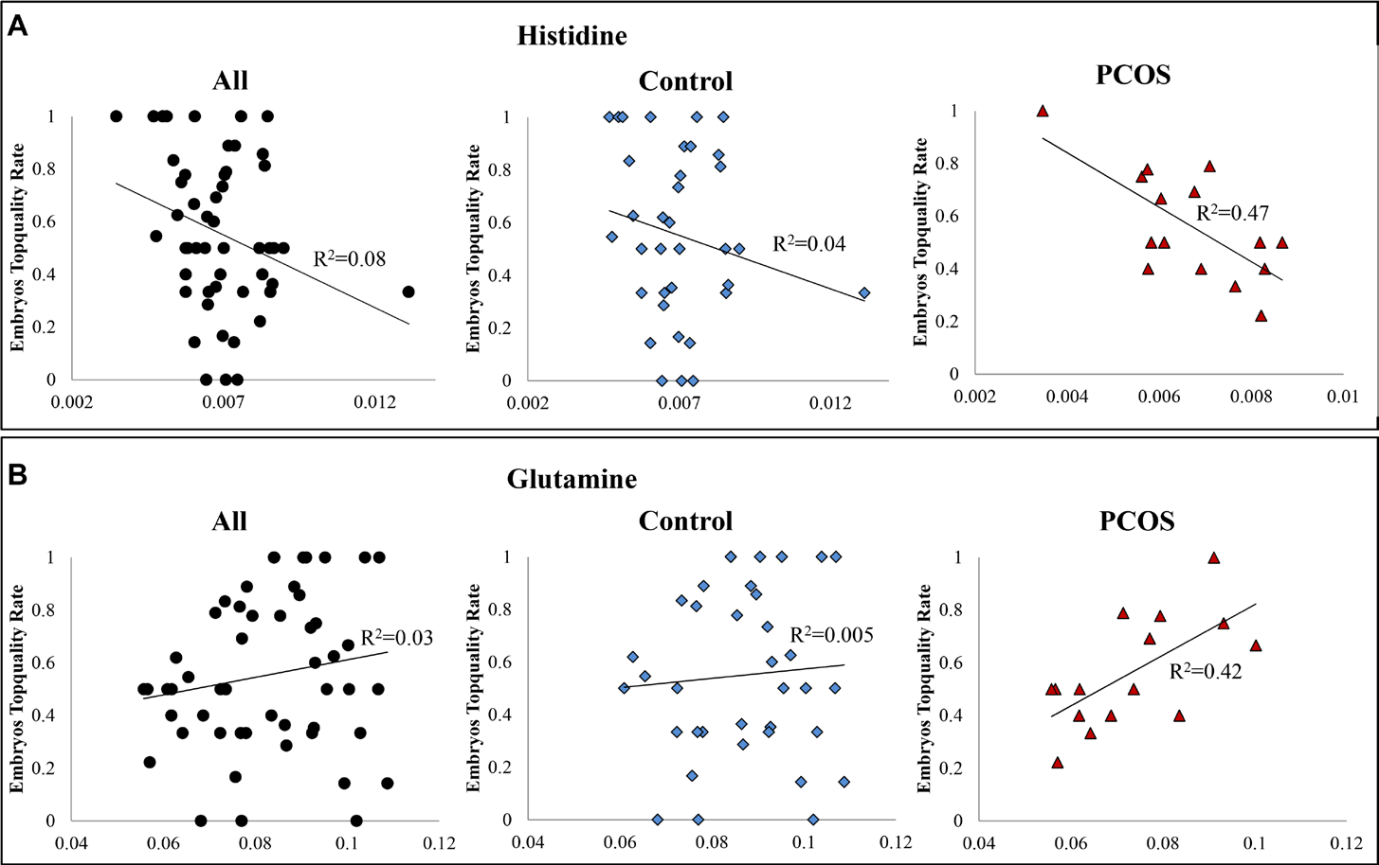
(**A**) Scatter plot of embryos top-quality rate and histidine. A significantly negative correlation exists between intrafollicular histidine level and embryos top-quality rate in PCOS patients (y = −103.36× + 1.25, *R*^2^ = 0.47, *p* < 0.005); (**B**) Scatter plot of embryos top-quality rate and glutamine. A significantly positive correlation exists between intra-follicular glutamine level and embryos top-quality rate in PCOS patients (y = 9.62× − 0.14, *R*^2^ = 0.42, *p* < 0.009). X-axis was abundance of the metabolite in NMR spectra (integral of peak region); y-axis was the embryos top-quality rate.

Creatine, leucine, isoleucine, and tyrosine were highly negatively correlated with embryos quality in all samples (Supplementary Figure 5). While, the correlations were weakened in PCOS sample cohort. In comparison, histidine exhibited highly negative correlation and glutamine presented highly positive correlation with embryos top-quality rate respectively only in PCOS patients (Figure 4). The ANOVA suggested significant linear relationship between embryos top-quality rate and both metabolites respectively. All these observations indicated that PCOS induced metabolic perturbations affect ART process.

## DISCUSSION

PCOS is a heterogeneous disorder with different phenotypes in affected women. In this study, we are the first to apply NMR to investigate changes of metabolic profile in PCOS follicular fluids. The changes in the levels of follicular metabolites reflected abnormal metabolic pathway fluctuation that related to physiological functions. Moreover, it is important to explore the intra-follicular metabolites pattern because PCOS patients undergoing IVF always contain morphologically normal metaphase II oocytes but have impaired oocyte development [19].

Different from the findings of metabolites in PCOS plasma [11, 13], pyruvate and lactate were significant decreased, together with increased level of acetate in follicular fluids of PCOS patients. This indicated the alteration in pyruvate metabolism and glycolysis, which were highlighted by the impact value of 0.42 and 0.1 respectively in Figure 2A. It suggested impaired glucose mechanism were found in granulosa-lutein cells from women with polycystic ovaries [20]. The abnormal insulin level contributes to the decrease of glucose uptake and related metabolism in PCOS ovary and follicles. Central insulin resistance could be one but not the only reason of hyperinsulinemia in PCOS [21]. Those regulators affecting insulin functions are also unusually expressed in PCOS ovaries, for example, insulin resistance associated phosphatidylinositol 3-phosphate kinase (PI3-K) is down-regulated. Similarly, the down-regulated annexin A2 suppresses the expression of glucose transporter GLUT-4, leading to the deficiency of glucose transportation [22]. It is also supported by other studies that some involving pathways like glycolysis which have been found down-regulated in endometrium of PCOS women [23]. From our study, although the abnormal glucose level was not observed in PCOS follicular fluids, a significantly negative correlation of glucose with both fertilization rate and cleavage rate were found in PCOS. It is also suggested by other studies that, the fertilization [24] and cleavage [25] steps are quite glucose uptake dependent.

A slightly increase of acetoacetate and 3-hydroxy-butyrate was observed in PCOS follicular fluids at meanwhile. These ketone body products come from the up-regulated fatty acid degradation. It is probably that alternative energy pathways were provoked to compensate the impairment of pyruvate metabolism and glycolysis for energy demanding [7]. The acetoacetate and 3-hydroxy-butyrate were also positively correlated with 2PN fertilization rate in PCOS patients. While, this significant correlation was not found in control group. These ketone bodies were crucial as energy substrates in early embryo development [26]. It further supported that the energy alternatives were raised because of PCOS.

Similarly, significant decrease of alanine and glutamine, as well as a slight decrease of tyrosine in PCOS follicular fluids demonstrated an alteration in amino acid metabolism. It is likely that amino acids were increasingly consumed to provide energy. However, glutamine was found to be positively correlated with embryos top-quality rate only in PCOS. This was agreed with that glutamine and lactate were found positively correlated, and lactate was decreased in oocytes fertilization and cleavage steps [25]. It may indicate, with deficient energy source, the amount of energy alternative was crucial for embryo development.

It is not surprising that a significantly increased level of glycerol and lipid region (Pk18), cholesterol and a slightly higher level of lipids (LDL) were present in PCOS follicular fluids. It was the same with the findings in PCOS serum from both NMR and MS studies, in which levels of other free fatty acids like palmitoleic acid, linoleic acid etc. raised too [21]. These are typical signs of dyslipidemia, which characterized by a decrease in HDL, an increase in LDL and triglycerides in PCOS. This is due to the impaired lipase expression and altered lipolysis caused by insulin resistance [27].

Glycoprotein was significantly increased in PCOS follicular fluids. This was in the same trend as observed in PCOS sera [11]. Evidence has been shown that glycoprotein is closely related to various inflammatory disorders, including infection, cardiovascular disorder, diabetes, etc [28]. The finding in our study indicated oocytes’ environment may be related to low-grade inflammation in PCOS patients. Such intra-follicular inflammation was also found in PCOS patients from other studies. The altered GC expression of cytokines, chemokines, and immune cell biomarkers is possibly the main cause [29].

Beside, an interesting finding was that, in all follicular fluid samples, significantly negative correlations appeared between embryos top-quality rate and several amino acids, including creatine, leucine, isoleucine, as well as tyrosine. The correlations with first three amino acids were even significant in control group. Although not known by underlying mechanism, it is in agreement with a study that 11 amino acids (including isoleucine, etc.) were found elevated in follicular fluids from patients with repeated IVF failure [30].

Despite metabolic difference that was observed in PCOS follicular fluid, as well as interesting findings that explained the correlation between follicular fluid metabolites and ART outcomes, some limitations needed to be addressed in the study. Firstly, the relatively small number of available samples in this pilot study may limit the statistical power to discovery small changes in the metabolic profile. Further validation of different sample cohorts will be expected to draw a clearer picture. In addition, this exploratory study limited the investigation on small molecules with relatively high contents detectable by NMR. A deeper observation using more sensitive techniques will be expired in future studies.

## MATERIALS AND METHODS

### Follicular fluid samples

Human follicular fluid samples were obtained from the Renmin Hospital of Wuhan University. The study was approved by the Institutional Review Boards. All 15 PCOS patients were diagnosed according to the criteria from both the European Society of Human Reproduction and Embryology and the American Society for Reproductive Medicine in 2003 [31]. The 36 healthy controls were from the women under reproductive age who participated in ART because of their couples’ sterility. All individuals from both sample cohorts were Chinese females and from the same province (Hubei) in China, which means they had the similar diet and life style. All the samplings were carried out in the mornings, and appropriate diet was suggested before oocyte retrieval. The same ovarian stimulation protocol was applied. Oocytes were retrieved using transvaginal ultrasound under guidance 34–36 hours after HCG administration. Oocytes were collected. Follicular fluid from same patient was combined, transported under dry ice and stored at −80°C until analysis.

### Clinical information

In 3 hours after oocyte retrieval, insemination was conducted with Percoll-prepared spermatozoa, whose population of motile spermatozoa was more than 7000–10000/ml. Subsequently, oocytes were transferred to a fresh medium. The occurrence of 2 pronuclei (PN) was observed and the number was recorded. The 2PN fertilization rate was calculated by dividing 2PN counts by oocyte retrieved number. After syngamy, the ratio of cleavage number to the fertilization number was calculated as cleavage rate. The specific evaluation standard for embryo quality on 3 days after cleavage was as follows: grade I, embryos with equivalent size and shape, and no obvious fragments were found; grade II, with slightly different size and shape, the percentage of DNA fragment was less than 20%; grade III, with significantly different size and the DNA fragment was between 20% and 50%; grade IV, DNA fragment was more than 50%. Embryos with good quality were defined as the embryos on day 3 belong to grade I or II, and the number of blastomeres were 6 to 9. Finally, the top-quality embryo rate was calculated as the ratio of good quality embryo number to the 2PN cleavage number.

### NMR experiments

Each frozen follicular fluid sample was thawed, vortexed, centrifuged and then 530 μL was transferred to 5-mm NMR tubes. A co-axial capillary containing 60 μL TSP (0.53 mmol) in D2O was placed into the NMR tube to serve as chemical shift and quantitative reference. The samples were randomized before performing the NMR experiments. All ^1^H NMR experiments were carried out at a 25^°^C on a Bruker DRX500-MHz NMR spectrometer. ^1^H NMR data for each sample was acquired using CPMG (Carr-Purcell-Meiboom-Gill) pulse sequences. Water was suppressed using a presaturation pulse. For each spectrum, 64 transients were collected and 16K data points were acquired using a spectral width of 6000 Hz. An exponential weighting function corresponding to 0.5 Hz line broadening was applied to the free-induced decay before Fourier transformation. Phasing and baseline correction were applied using Bruker Xwinnmr software version 3.5.

### Data analysis

The NMR spectra obtained using the CPMG sequence were devoid of broad peaks from macromolecules and hence were more suitable for investigating altered levels of metabolites. The datasets were aligned with reference to the alanine peak at 1.46 ppm using MestReNova version 6.6.1. Each CPMG NMR spectrum was binned to 4 k frequency buckets of equal size (0.003 ppm). The spectra were then normalized with reference to the total spectra integral (δ 10.0 ∼0.40 ppm), excluding the residual water and urea region δ 4.7 ∼ 5.2 ppm. The spectral data were initially mean-centered and subjected to supervised multivariate statistical analysis including partial least-squares analysis (PLS) and orthogonal-signal-corrected partial least-squares (OSC-PLS) analysis.

In a more targeted analysis, a total of 59 representative peak regions were identified according to the Human Metabolome Data Base and the comprehensive NMR study of follicular fluid [32]. Integrals for these peak regions were obtained and standardized to a sample mean of 0 and standard deviation of 1 to avoid any magnitude influences. Subsequently, these 59 metabolite signals were subjected multivariate analysis, Student's *t*-test, and correlation analysis with ART clinical data using Matlab (R2008a; Mathworks, Natick, MA) and PLS Toolbox (version 4.11, Eigenvector Research Inc.). The heat map and ANOVA result of linear regression fitting was constructed by R (version 3.3.1).

## CONCLUSIONS

The present study is a pioneer to investigate metabolic variations in PCOS follicular fluid based on NMR metabolomics approach. The differential metabolites indicated dyslipidemia and main alternations in pyruvate metabolism, glycolysis, amino acid metabolism, and inflammation. The findings provided deep insight for PCOS pathogenesis mechanisms that happened in oocytes environment. Furthermore, the metabolic effects on oocytes quality were firstly explored based on the ART results, which suggested embryo top-quality rate was closely correlated with creatine, leucine and isoleucine in all follicular fluids, while specifically related to histidine and glutamine only in PCOS patients. These results provided us a better knowledge of human oocyte physiology, and metabolic effects on oocyte quality. Future work would be expected to increase the sample number for further validation, and to explore more subtle metabolic variation using techniques with higher sensitivity.

## SUPPLEMENTARY MATERIALS FIGURES AND TABLES


