# Supplementary material for: Follicular metabolic changes and effects on oocyte quality in polycystic ovary syndrome patients
Source: Oncotarget. 2017 Jul 6;8(46):80472–80. doi: 10.18632/oncotarget.19058 (PMC5655213; doi:10.18632/oncotarget.19058)
Supplement: Supplementary file 1 [file oncotarget-08-80472-s001.pdf]

Follicular metabolic changes and effects on oocyte quality in polycystic ovary syndrome patients

Supplementary Materials

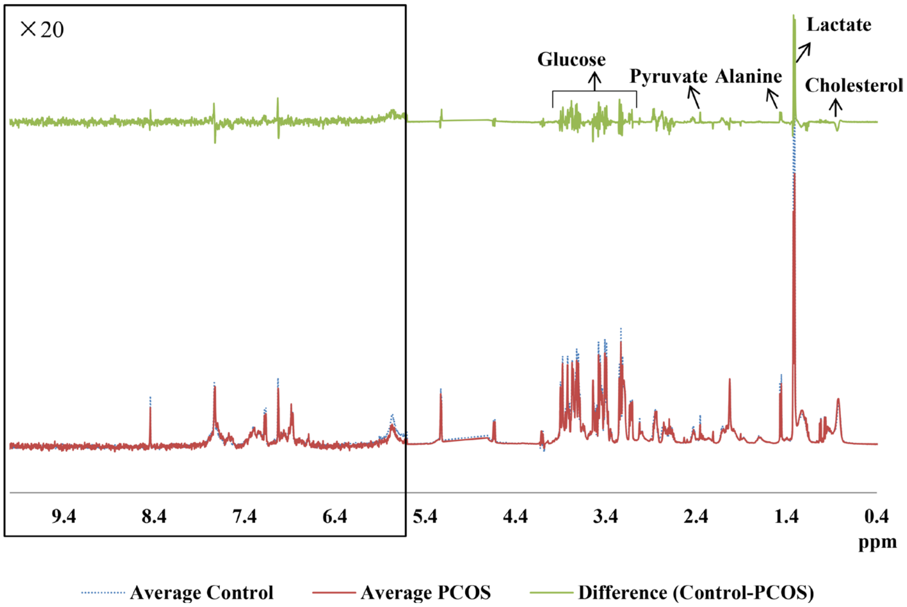

Supplementary Figure 1: ^{1}H NMR mean spectra of CRC and healthy serum (bottom), and the difference spectrum (top).

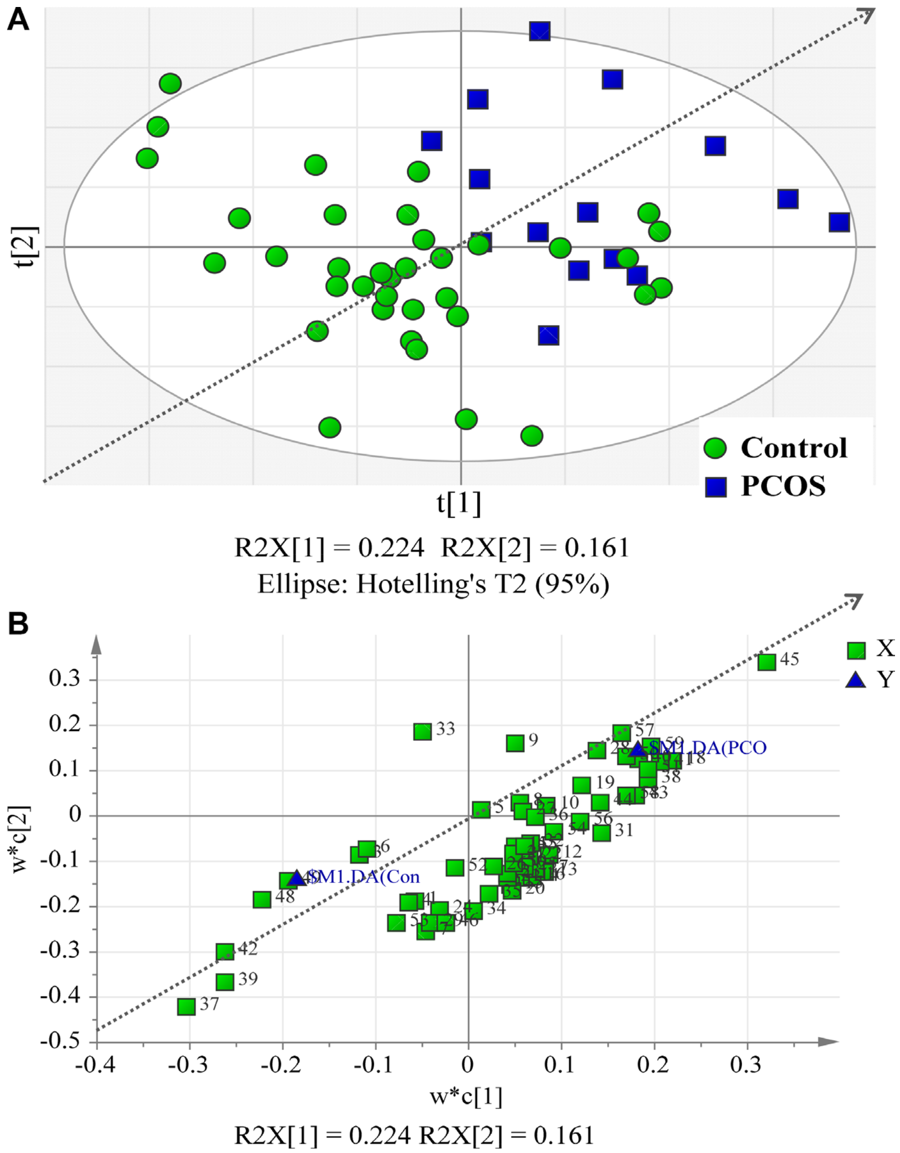

Supplementary Figure 2: (**A**) PLS score plot based on 59 metabolite regions of follicular fluid NMR spectra. A dash line with arrow illustrated the group separation direction; (**B**) PLS loadings plot based on 59 metabolite regions of follicular fluid NMR spectra. The numbers represent peak IDs. Acetate (PK45), glutamine (PK37, 42), and pyruvate (PK39) contributed most to the clustering.

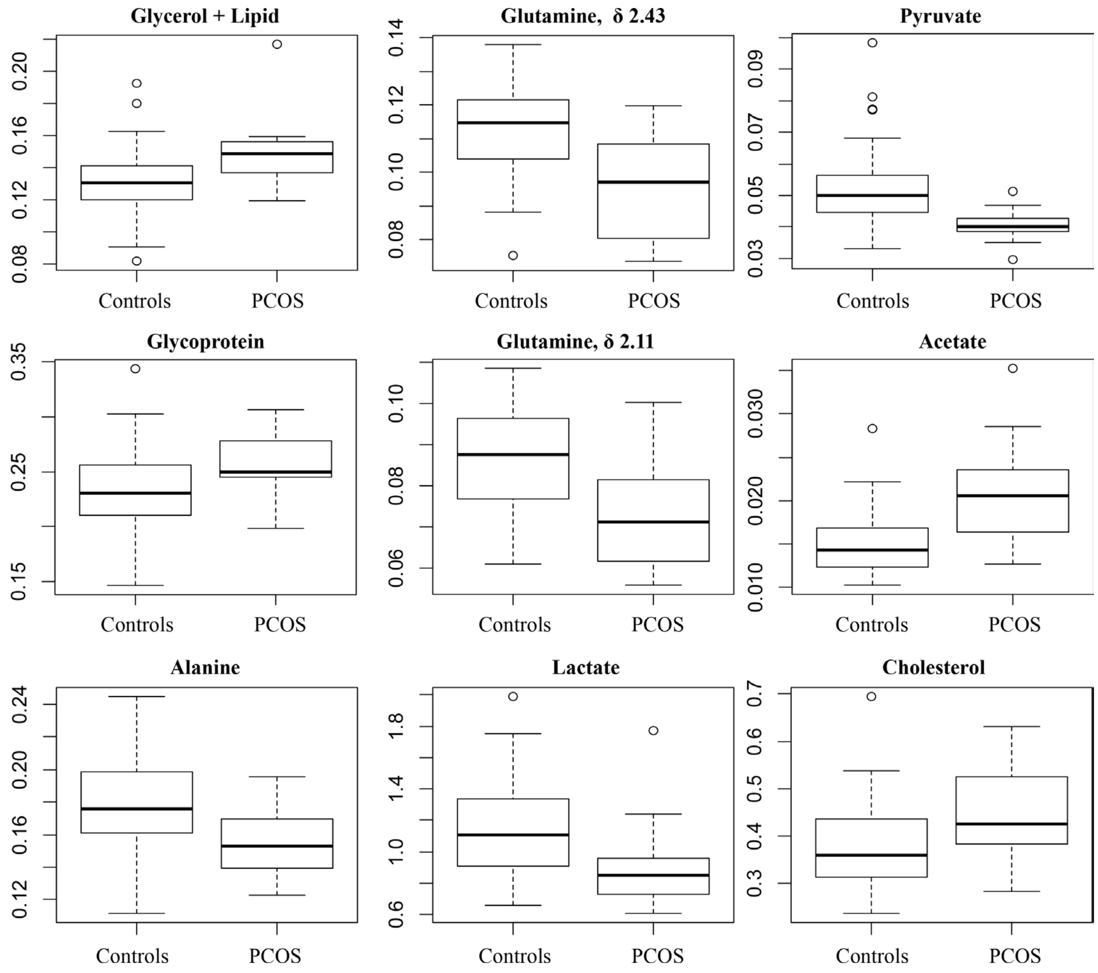

Supplementary Figure 3: Box-plots for nine metabolite regions found significantly different in PCOS follicular fluid.

c

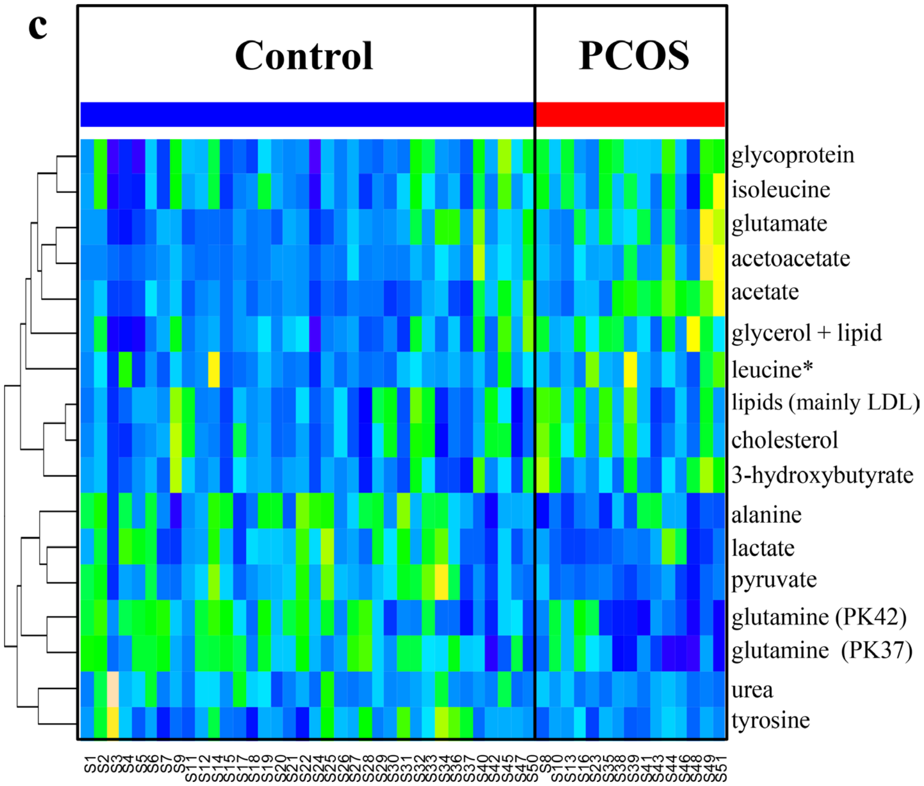

Supplementary Figure 4: Heat map of differential metabolites regions with *p* < 0.1.

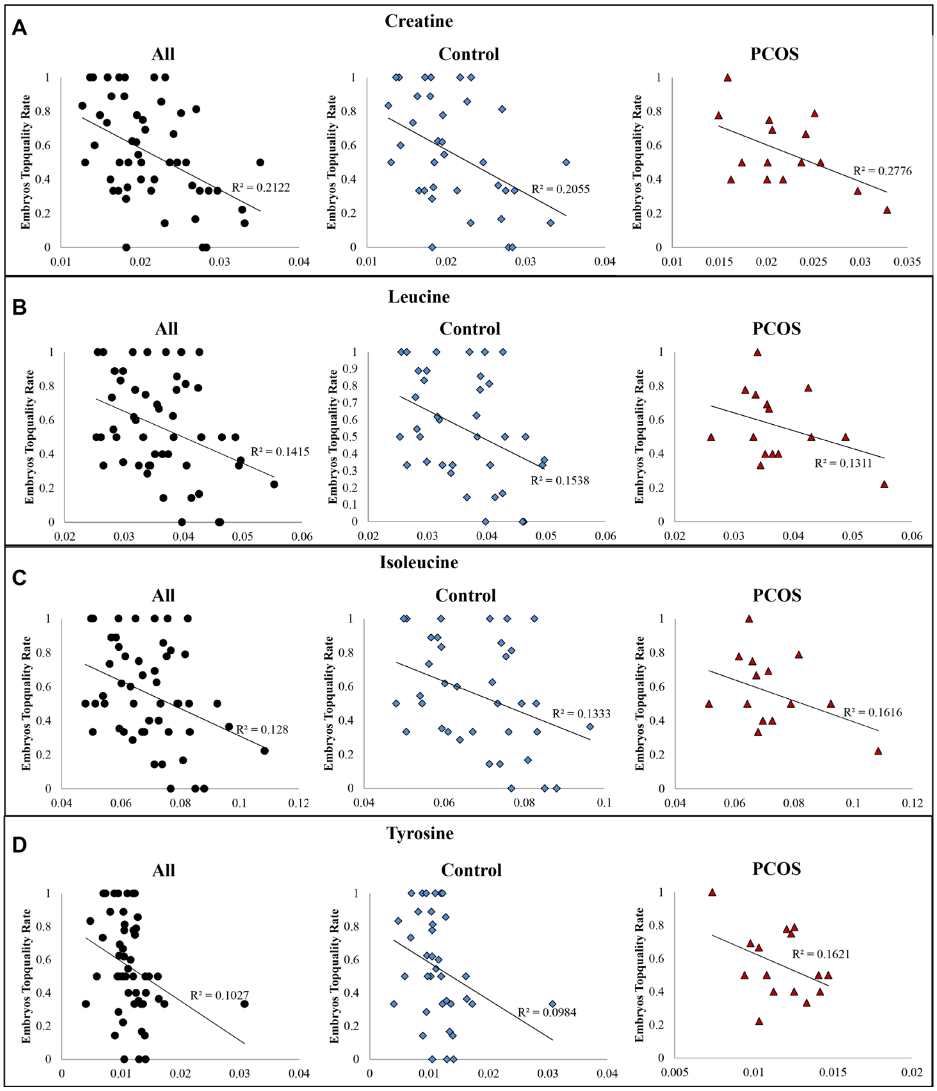

Supplementary Figure 5: (**A**) Scatter plot of embryos top-quality rate and creatine. A significantly negative correlation exists between intrafollicular creatine level and embryos top-quality rate in all samples; (**B**) scatter plot of embryos top-quality rate and leucine. A significantly negative correlation exists between intrafollicular leucine level and embryos top-quality rate in all samples; (**C**) scatter plot of embryos top-quality rate and isoleucine. A significantly negative correlation exists between intrafollicular isoleucine level and embryos top-quality rate in all samples; (**D**) scatter plot of embryos top-quality rate and tyrosine. A significantly negative correlation exists between intrafollicular tyrosine level and embryos top-quality rate in all samples.

Supplementary Table 1: Clinical information of the follicular fluids included in the study

	Healthy Controls	PCOS
No. of samples (patients)	36 (36)	15 (15)
Age (years)	31.9 ± 3.8	27.6 ± 3.0
Ethnicity	Chinese	Chinese
2PN fertilization rate	0.61 ± 0.24	0.55 ± 0.19
Cleavage rate	0.95 ± 0.12	0.94 ± 0.12
Top-quality embryos rate	0.55 ± 0.32	0.56 ± 0.21

Supplementary Table 2: Integrated 59 metabolite regions with *P*-value from Student *t*-test if less than 0.1

No.	Metabolite	Chemical shift (ppm)	Multiplicity	*p*-value	No.	Metabolite	Chemical shift (ppm)	Multiplicity	*p*-value
**Pk 1**	formate	[8.453, 8.438]	s		**Pk 31**	choline	[3.225 , 3.211]	s	
**Pk 2**	histidine	[7.751, 7.718]	s		**Pk 32**	phosphocholine	[3.195 , 3.181]	s	
**Pk 3**	tyrosine	[7.187, 7.154]	m	6.4 E-2	**Pk 33**	phenylalanine	[3.151 , 3.097]	dd	
**Pk 4**	histidine	[7.041, 7.015]	s		**Pk 34**	creatinine	[3.038 , 3.019]	s	
**Pk 5**	tyrosine	[6.919, 6.848]	m		**Pk 35**	creatine	[3.018 , 3.005]	s	
**Pk 6**	urea	[5.842, 5.684]	br. s.	6.7 E-2	**Pk 36**	citrate	[2.549 , 2.491]	d	
**Pk 7**	α-glucose	[5.246, 5.206]	d		**Pk 37**	glutamine	[2.463 , 2.402]	m	1.6 E-3
**Pk 8**	β-glucose	[4.656, 4.616]	d		**Pk 38**	glutamate	[2.401 , 2.367]	m	6.7 E-2
**Pk 9**	lactate	[4.121, 4.090]	q		**Pk 39**	pyruvate	[2.367 , 2.346]	s	2.8 E-5
**Pk 10**	creatinine	[4.049, 4.034]	s		**Pk 40**	3-hydroxuybutyrate	[2.345 , 2.310]	m	6.0 E-2
**Pk 11**	creatine	[3.927, 3.914]	s		**Pk 41**	acetoacetate	[2.226 , 2.204]	s	7.8 E-2
**Pk 12**	β-glucose	[3.914, 3.894]	dd		**Pk 42**	glutamine	[2.139 , 2.101]	m	3.7 E-3
**Pk 13**	β-glucose	[3.894, 3.867]	dd		**Pk 43**	glycoprotein	[2.047 , 2.005]	s	2.0 E-2
**Pk 14**	glucose	[3.858, 3.840]	dd		**Pk 44**	proline	[2.004 , 1.965]	m	
**Pk 15**	α-glucose	[3.840, 3.811]			**Pk 45**	acetate	[1.916 , 1.900]	s	2.5 E-3
**Pk 16**	glucose	[3.716, 3.693]	t		**Pk 46**	arginine*	[1.900 , 1.835]	m	
**Pk 17**	glucose	[3.693, 3.677]			**Pk 47**	lysine*	[1.751 , 1.646]	m	
**Pk 18**	glycerol + lipid	[3.674, 3.618]	m	1.1 E-2	**Pk 48**	alanine	[1.492 , 1.441]	d	3.5 E-3
**Pk 19**	glycine*	[3.552, 3.527]	s		**Pk 49**	lactate	[1.348 , 1.287]	d	1.9 E-2
**Pk 20**	α-glucose	[3.527, 3.507]	dd		**Pk 50**	lipids (mainly LDL)	[1.280 , 1.202]	–	6.4 E-2
**Pk 21**	glucose	[3.507, 3.493]	s		**Pk 51**	3-hydroxybutyrate	[1.202 , 1.174]	d	5.2 E-2
**Pk 22**	β-glucose	[3.492, 3.475]			**Pk 52**	valine	[1.042 , 1.008]	d	
**Pk 23**	glucose	[3.474, 3.458]			**Pk 53**	isoleucine	[1.006 , 0.994]		
**Pk 24**	glucose	[3.457, 3.431]			**Pk 54**	isoleucine	[0.954 , 0.953]		
**Pk 25**	glucose	[3.431, 3.419]			**Pk 55**	leucine	[0.953 , 0.940]	d	
**Pk 26**	glucose	[3.400, 3.381]	t		**Pk 56**	leucine	[0.940 , 0.929]	d	
**Pk 27**	glucose	[3.380, 3.364]			**Pk 57**	leucine*	[0.929 , 0.915]		9.9 E-2
**Pk 28**	TMAO	[3.360, 3.343]	s		**Pk 58**	isoleucine	[0.914 , 0.899]		7.6 E-2
**Pk 29**	β-glucose	[3.262, 3.244]			**Pk 59**	cholesterol	[0.871 , 0.771]	m	2.8 E-2
**Pk 30**	β-glucose	[3.244, 3.225]							

*The peaks could be overlapped with other metabolites.
